# The Use of Travel as an Appeal to Motivate Millennial Parents on Facebook to Get Vaccinated Against COVID-19: Message Framing Evaluation

**DOI:** 10.2196/43720

**Published:** 2023-08-01

**Authors:** Suraj Arshanapally, Tiearra Starr, Lauren Blackmun Elsberry, Robin Rinker

**Affiliations:** 1 National Center for Emerging and Zoonotic Infectious Diseases Centers for Disease Control and Prevention Atlanta, GA United States; 2 Cherokee Federal Tulsa, OK United States

**Keywords:** COVID-19, coronavirus, vaccination, travel, parents, millennial, appeal, health communication, social media, Facebook, infectious disease, message testing, public health, messaging, parenting, program

## Abstract

**Background:**

In summer 2021, the Centers for Disease Control and Prevention recommended that people get fully vaccinated against COVID-19 before fall travel to protect themselves and others from getting and spreading COVID-19 and new variants. Only 61% of parents had reported receiving at least 1 dose of the COVID-19 vaccine, according to a Kaiser Family Foundation study. Millennial parents, ages 25 to 40 years, were a particularly important parent population because they were likely to have children aged 12 years or younger (the age cutoff for COVID-19 vaccine eligibility during this time period) and were still planning to travel. Since Facebook has been identified as a popular platform for millennials and parents, the Centers for Disease Control and Prevention’s Travelers’ Health Branch determined an evaluation of public health messages was needed to identify which message appeals would resonate best with this population on Facebook.

**Objective:**

The objective was to evaluate which travel-based public health message appeals aimed at addressing parental concerns and sentiments about COVID-19 vaccination would resonate most with Millennial parents (25 to 40 years old) using Facebook Ads Manager and social media metrics.

**Methods:**

Six travel-based public health message appeals on parental concerns and sentiments around COVID-19 were developed and disseminated to millennial parents using Facebook Ads Manager. The messages ran from October 23, 2021, to November 8, 2021. Primary outcomes included the number of people reached and the number of impressions delivered. Secondary outcomes included engagements, clicks, click-through rate, and audience sentiments. A thematic analysis was conducted to analyze comments. The advertisement budget was evaluated by cost-per-mille and cost-per-click metrics.

**Results:**

All messages reached a total of 6,619,882 people and garnered 7,748,375 impressions. The *Family* (n=3,572,140 people reached, 53.96%; 4,515,836 impressions, 58.28%) and *Return to normalcy* (n=1,639,476 people reached, 24.77%; 1,754,227 impressions, 22.64%) message appeals reached the greatest number of people and garnered the most impressions out of all 6 message appeals. The *Family* message appeal received 3255 engagements (60.46%), and the *Return to normalcy* message appeal received 1148 engagements (21.28%). The *Family* appeal also received the highest number of positive post reactions (n=82, 28.37%). Most of the comments portrayed negative opinions about COVID-19 vaccination (n=46, 68.66%). All 6 message appeals were either on par with or outperformed cost-per-mille benchmarks set by other similar public health campaigns.

**Conclusions:**

Health communicators can use travel, specifically the *Family* and *Return to normalcy* message appeals, to successfully reach parents in their future COVID-19 vaccination campaigns and potentially inform health communication messaging efforts for other vaccine-preventable infectious disease campaigns. Public health programs can also utilize the lessons learned from this evaluation to communicate important COVID-19 information to their parent populations through travel messaging.

## Introduction

### Background

In summer 2021, the Centers for Disease Control and Prevention (CDC) recommended that people get fully vaccinated against COVID-19 before fall travel to protect themselves and others from getting and spreading COVID-19 and new variants [1,2]. At that time, COVID-19 vaccines were only available to people over the age of 12 years, and vaccination rates were slowing. Only 61% of parents reported receiving 1 dose of a COVID-19 vaccine, which was lower than the 71% among adults without children [3]. While 62% of parents reported concerns about exposing their loved ones to COVID-19 and 64% were concerned about returning to normal activities in public such as using public transport, 60% of parents still planned on traveling in the fall [4].

Among those parents still planning on traveling for fall 2021, overall, 26% were planning a trip with someone 12 years or older who was unvaccinated, and 16% were planning to travel with a child who was not yet eligible for COVID-19 vaccination. In addition, only 41% of the unvaccinated parents planning travel indicated that they planned to get vaccinated before their travel [4].

Among parents, millennial parents were a population of particular interest because they were likely to have children 12 years or younger—the age cutoff for COVID-19 vaccine eligibility during that time period [5]. According to a Kaiser Family Foundation study conducted from July 15, 2021, to August 2, 2021, only 49% of parents aged 18 to 39 years had received at least 1 dose of the COVID-19 vaccine [3]. Furthermore, Kaiser Family Foundation found that parental vaccination intentions for their children were correlated with their own vaccination status. For instance, 61% of parents who received at least 1 dose of the COVID-19 vaccine stated that their children, ages 12 to 17 years, also received at least 1 dose of the COVID-19 vaccine, compared to only 4% of unvaccinated parents. Therefore, it was important to identify messaging that could motivate millennial parents to get vaccinated to protect themselves and their unvaccinated children during travel.

Studies performed by the Pew Research Center identified Facebook as a popular platform among millennials and parents [6,7]. Additionally, a recent paid digital marketing campaign conducted by the CDC, Arshanapally et al [8] demonstrated that Facebook serves as an effective platform to reach parents of young children with public health messages.

Throughout the pandemic, government and public health agencies have used social media to communicate vital health information, such as the importance of getting vaccinated against COVID-19. However, a sizeable portion of the population in the United States were still not convinced of the need to get vaccinated [9], including 56% of unvaccinated parents who planned to wait until they felt comfortable with the COVID-19 vaccine [10].

Chou and Budenz [11] highlight the effectiveness of using emotional appeals to communicate the importance of getting vaccinated against COVID-19. They mention appeals that spark positive emotions such as hope and joy as well as counter negative emotions such as anxiety and fear. Due to the ongoing travel behaviors and low vaccination rates among millennial parents [3,4] throughout the COVID-19 pandemic, the CDC’s Travelers’ Health Branch determined an evaluation of public health messages was needed to identify which message appeals would resonate best with this population on Facebook.

### Objective

The objective was to evaluate which travel-based public health message appeals aimed at addressing which parental concerns and sentiments about COVID-19 vaccination would resonate most with millennial parents (25 to 40 years old) using Facebook Ads Manager, developed by Meta, and social media metrics.

## Methods

### Project Design

The project had an advertising budget of US $26,600 to test 6 distinct travel-based public health message appeals about getting vaccinated against COVID-19 before travel with US-based millennial parents, aged 25 to 40 years, who had an interest in travel. The messages were disseminated from October 23, 2021, to November 8, 2021.

Messages were disseminated using Facebook Ads Manager, a paid advertising management service used to oversee paid social media messages across the Facebook platform. Messages were displayed on Facebook news feeds, video feeds, on Explore pages, and more [12]. There were multiple options available on Facebook to target the desired audience. For this project, the target audience was defined as US-based parents between the ages of 25 to 40 years who are frequent travelers. On Facebook, the audience segment called “Frequent Travelers” are identified as people who list travel and vacation as interests or regularly engage with travel content.

Facebook Ads Manager required ads to contain a business objective, also referred to as a “Result.” Because the project aimed to test which appeal was most successful at exposing the COVID-19 vaccination message to the target audience, the total number of people reached, or Reach, was identified as the desired Result. The Reach metric’s performance can be affected by data sampling, bid strategy, budget, audience targeting, and audience engagement [13-15]. The bid strategy selected was goal-based bidding, which meant that the total advertising budget was averaged across the campaign lifespan; however, Facebook will also dynamically bid as high as needed to maximize results based on the campaign goal. Facebook’s feature, *Campaign Budget Optimization* (CBO), allocated more of the campaign budget to the top-performing ads and less to low-performing ads based on criteria within Facebook’s algorithm [16]. By using this feature, each dollar was spent optimally based on the data-driven targeting features of Facebook’s algorithm. If the CBO feature was not used and funding was equally distributed to each message appeal, then a significant portion of the funding would have been used to deliver less effective message appeals.

### Message Development

**Figure 1:**
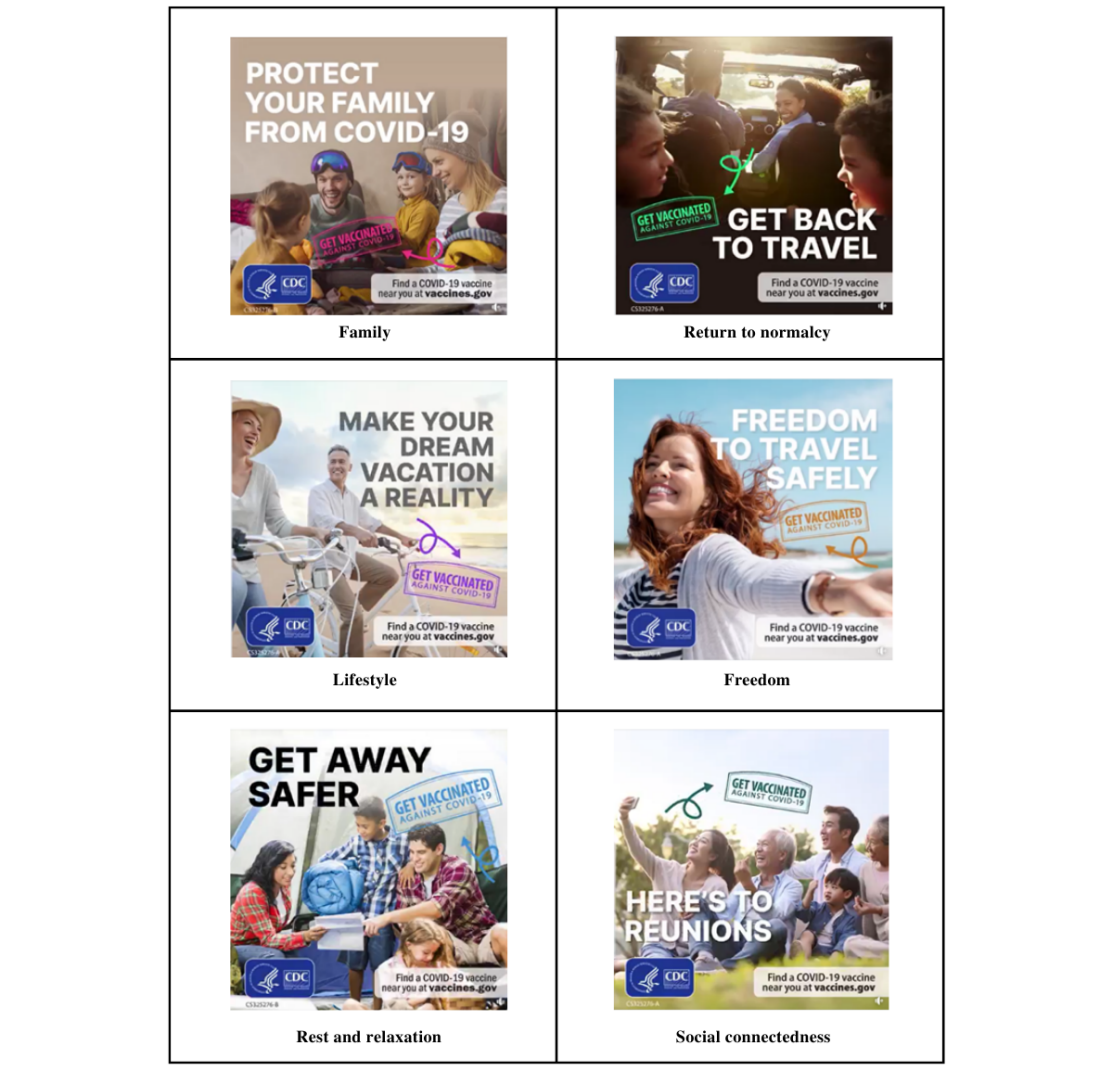
Visual components of Message appeals.

The following 6 unique message appeals were analyzed: *Family*, *Return to normalcy*, *Lifestyle*, *Freedom*, *Rest and relaxation*, and *Social connectedness*. All were informed by insights from a Harris Poll survey conducted in fall 2021 (Table 1). Each message appeal included a clear and accessible text component designed using the CDC Plain Language framework [17]. Message appeals also included stock images formatted as graphics interchange formats. Stock images were used to provide context to the appeal. All stock images included people participating in various travel-related activities. The images were treated with a similar design overlay and included the CDC logo. Each image contained a short, specific tagline and the same call-to-action message (“Find a COVID-19 vaccine near you at vaccines.gov”), with a short text component as part of the Facebook post. Screenshots of the 6 message appeals can be found in Figure 1.

**Table 1:** Message appeal development.

Insights, The Harris Poll [10]	Appeal	Text component
78% of parents state that they miss gatherings with friends and family during the COVID-19 pandemic.	Family	*Planning a family vacation? Keeping your kids healthy starts with you. Get your family fully vaccinated against COVID-19 before your trip.*
60% of parents indicate concerns about returning to normal activities.	Return to normalcy	*Wondering how your family can get back to traveling safely this fall? The first step is to get your family fully vaccinated against COVID-19.*
When parents were asked what they are planning to purchase once things return to normal, 34% said hotel stays and 32% said plane tickets.	Lifestyle	*Thinking about planning your dream vacation? Get your family fully vaccinated against COVID-19 and make your dream a reality.*
28% of parents feel claustrophobic since they are unable to escape their homes and 29% feel annoyed by the lack of personal space and the inability to get away from their families.	Freedom	*Getting your family fully vaccinated against COVID-19 is your ticket to freedom. Get vaccinated before travel.*
34% of parents feel overwhelmed with the task of trying to balance work at home and other needs of their families.	Rest and relaxation	*Parents, do you need a break from your daily routine? Getting your family fully vaccinated against COVID-19 is your ticket to a relaxing vacation.*
67% of parents state that they miss going to social gatherings during the pandemic when many restrictions have been placed.	Social connectedness	*Get your family fully vaccinated against COVID-19 before your travel and safely reconnect with loved ones near and far.*

### Data Collection

Social media metrics used are defined in Textbox 1. The Reach and Impressions metrics were used to evaluate how successful each message appeal was at exposing the COVID-19 vaccination message to the target audience. The Engagements, Post reactions, Post shares, and Comment metrics provided insight into how the target audience connected with each message appeal.

**Definitions of social media metrics.**

- Result (Reach): reach was defined as the business objective, or Result, on Facebook. Reach is the number of people who saw a message appeal at least once.
- Impressions: number of times a message appeal is served to a user (includes repeat exposures).
- Engagements: number of actions (reactions, shares, comments, and clicks) taken upon seeing the message appeal.
- Post reactions: specific type of engagement that allows users to interact with a message appeal based on sentiment. Post reactions include the ability to express like, love, care, laughter, surprise, sadness, and anger
- Post shares: specific type of engagement that allows users to distribute the message appeal with their networks.
- Comments: written remarks expressing opinions or reactions to the message appeal.
- Link clicks: the number of clicks on the message appeal directing users to www.vaccines.gov.
- Click-through rate: total number of clicks on the message appeal divided by the total number of impressions.
- Cost-per-mille: total advertising budget spent per 1000 impressions. It is calculated by dividing the total amount spent by number of impressions, and then multiplying by 1000.
- Cost-per-click: total advertising budget spent per message appeal divided by the number of link clicks to www.vaccines.gov.

Furthermore, the Post reactions metric was categorized into 3 sentiments: positive, neutral, and negative. Positive sentiment was defined as clicking on the following post reactions: Like, Love, or Care. Neutral sentiment, which included sentiments that were either unclear or neither positive nor negative, was defined as clicking on the following post reactions: Wow (Surprise) or Ha-Ha (Laughter). Negative sentiment was defined as clicking on the following post reactions: Sad or Anger. In addition, a thematic analysis was conducted on the Facebook comments. Two health communication specialists coded the comments to analyze the sentiment and any emerging themes. Similar to Post reactions, the sentiment was categorized as positive, neutral, or negative sentiment toward the COVID-19 vaccination message appeal. For the themes, observed and relevant patterns in the data about the COVID-19 vaccination or travel were noted. The Link clicks and Click-through rate (CTR) metrics demonstrated how successful the message appeal was at getting the target audience to visit the website: www.vaccines.gov. The cost-per-mille (CPM) and cost-per-click (CPC) metrics were used to evaluate how the advertising budget was spent to disseminate messages and get link clicks.

### Ethical Considerations

Institutional review board approval was not required for this project as no human participants were involved, and analyzed data were limited to publicly available digital metrics collected in aggregate.

## Results

### Reach

All messages reached a total of 6,619,882 people and garnered 7,748,375 impressions (Table 2). The *Family* and *Return to normalcy* message appeals reached the greatest number of people and garnered the most impressions out of all 6 message appeals. The *Family* message appeal was seen by 53.96% of all people reached (n=3,572,140 people reached) and delivered 58.28% of all impressions (n=4,515,836 impressions). The *Return to normalcy* message appeal was seen by 24.77% of all people reached (n=1,639,476 people reached) and delivered 22.64% of all impressions (n=1,754,227 impressions).

**Table 2:** Reach^a^ and Impressions^a^ for Message appeals.

Message appeals	Reach, n (%)	Impressions, n (%)
Family	3,572,140 (53.96)	4,515,836 (58.28)
Return to normalcy	1,639,476 (24.77)	1,754,227 (22.64)
Lifestyle	492,289 (7.44)	513,792 (6.63)
Freedom	398,986 (6.03)	418,823 (5.41)
Rest and relaxation	312,104 (4.71)	321,612 (4.15)
Social connectedness	204,887 (3.10)	224,085 (2.89)
Total	6,619,882 (100)	7,748,375 (100)

^a^See Textbox 1 for definitions.

### Engagement

The 6 message appeals were engaged with a total of 5384 times (Tables 3-5). Among the total engagements, there were 13 post shares, 67 comments, 289 post reactions, and 5025 link clicks. The *Family* message appeal garnered 60.46% (n=3255 engagements) of all engagements. Among these engagements, this appeal received 7 post shares (53.85%), 37 comments (55.22%), 162 post reactions (56.06%), and 3049 link clicks (60.68%). The *Return to normalcy* message appeal garnered 21.28% (n=1148 engagements) of all engagements, the second highest number of engagements among all message appeals. Among these engagements, this appeal received 2 (15.38%) post shares, 7 (10.45%) comments, 44 (15.22%) post reactions, and 1095 (21.79%) link clicks.

**Table 3:** Engagements^a^, Post shares^a^, Comments^a^, Link clicks^a^, CTR^a^, and Post reactions^a^ for Message appeals.

Message appeals	Engagements, n (%)	Post shares, n (%)	Comments, n (%)	Link clicks, n (%)	CTR (%)	Post reactions, n (%)
Family	3255 (60.34)	7 (53.85)	37 (55.22)	3049 (60.68)	0.0675	162 (56.06)
Return to normalcy	1148 (21.28)	2 (15.38)	7 (10.45)	1095 (21.79)	0.0624	44 (15.22)
Lifestyle	333 (6.17)	1 (7.69)	2 (2.99)	310 (6.17)	0.0603	20 (6.92)
Freedom	318 (5.90)	1 (7.69)	9 (13.43)	286 (5.69)	0.0683	22 (7.61)
Rest and relaxation	205 (3.80)	1 (7.69)	7 (10.45)	183 (3.64)	0.0569	14 (4.84)
Social connectedness	135 (2.50)	1 (7.69)	5 (7.46)	102 (2.03)	0.0455	27 (9.34)
Total	5394 (100)	13 (100)	67 (100)	5025 (100)	0.0649	289 (100)

^a^See Textbox 1 for definitions.

**Table 4:** Positive^a^, Neutral^b^, and Negative^c^ post reactions (N=289) for Message appeals.

Message appeals	Positive, n (%)	Neutral, n (%)	Negative, n (%)	Total, n (%)
Family	82 (28.4)	73 (25.3)	7 (2.4)	162 (56.1)
Return to normalcy	32 (11.1)	11 (3.8)	1 (0.3)	44 (15.2)
Lifestyle	7 (2.4)	7 (2.4)	6 (2.1)	20 (6.9)
Freedom	8 (2.8)	13 (4.5)	1 (0.4)	22 (7.6)
Rest and relaxation	5 (1.7)	9 (3.1)	0 (0)	14 (4.8)
Social connectedness	5 (1.7)	13 (4.5)	9 (3.1)	27 (9.3)
Total	139 (48.1)	126 (43.6)	24 (8.3)	289 (100)

^a^Positive sentiment was defined as clicking on the following post reactions: Like, Love, or Care.

^b^Neutral sentiment, which also included ambiguous sentiments, was defined as clicking on the following post reactions: Wow (Surprise) or Ha-Ha (Laughter).

^c^Negative sentiment was defined as clicking on the following post reactions: Sad or Anger.

**Table 5:** Sentiments from Facebook comments (N=67).

Message appeals	Positive, n (%)	Neutral, n (%)	Negative, n (%)	Total, n (%)
Family	2 (3)	15 (22)	20 (30)	37 (55)
Return to normalcy	0 (0)	3 (5)	4 (6)	7 (11)
Lifestyle	0 (0)	0 (0)	2 (3)	2 (3)
Freedom	0 (0)	1 (2)	8 (12)	9 (13)
Rest and relaxation	0 (0)	0 (0)	7 (11)	7 (11)
Social connectedness	0 (0)	0 (0)	5 (8)	5 (8)
Total	2 (3)	19 (28)	46 (69)	67 (100)

### Clicks

The 6 message appeals received a total of 5025 link clicks with a CTR=0.0649% (Table 3). The *Family* and *Return to normalcy* message appeals received the greatest number of clicks. The *Family* message appeal garnered 60.68% of all clicks (n=3049 clicks; CTR=0.0675%). The *Return to normalcy* message appeal garnered 21.79% of all clicks (n=1095 clicks; CTR=0.0624%). The *Freedom* message appeal (n=286 clicks) had the highest CTR of 0.0683%, and the *Social connectedness* message appeal (n=102 clicks) had the lowest CTR of 0.0455%.

### Sentiment

Analysis of the Post reactions metric was used for the sentiment analysis. Of the 289 post reactions across all 6 message appeals (Table 4), there were 139 positive (Like, Love, and Care) reactions (48.1%), 126 neutral or ambiguous (Laughter and Surprise) reactions (43.6%), and 24 negative (Sadness and Anger) reactions (8.3%). The *Family* message appeal garnered the greatest number of positive reactions (n=82, 28.4%) and neutral or ambiguous reactions (n=73, 25.3%). The *Return to normalcy* message appeal also garnered a significant number of positive reactions (n=32, 11.1%). The *Social connectedness* message appeal received the largest number of negative post reactions (n=9, 3.1%), which included sadness and anger, among all 6 message appeals (Table 4).

The 67 comments posted across all 6 message appeals (Table 5) were coded to identify sentiments. The majority of the comments portrayed a negative sentiment toward the COVID-19 vaccine (n=46, 69%). Negative sentiment included mentions that the vaccine was “poison” and “experimental.” There were 19 (28%) comments that portrayed a neutral or ambiguous sentiment toward the COVID-19 vaccine. Neutral sentiment included genuine questions about how the COVID-19 vaccine works and comments that were neither pro- nor antivaccine. Few comments portrayed a positive sentiment toward the COVID-19 vaccine (n=2, 3%). Positive sentiment included a mention of getting vaccinated.

A thematic analysis was conducted to identify common themes among the comments (Table 6). A portion of comments spread misinformation about the COVID-19 vaccine (n=6, 9%), such as “no one under the age of 20 has died from Covid.” There were 13 comments (19%) that mentioned politics, such as “The Dems do not want real facts. They want sheep.”

**Table 6:** Themes from Facebook comments.

Themes and sample comments			Message appeal	
**Misinformation (n=6, 9%), comment shared inaccurate information regarding COVID-19 or the COVID-19 vaccine**				
	*Does anybody really trust the CDC vaccines are poisonous and experimental It will depopulate the earth.*	Family		
	*No one under the age of 20 has died from Covid take your vaccine and shove it.......what a bunch of BS.*	Family		
**Politics (n=13, 19%), comment referenced politics or politicians**				
	*The Dems do not want real facts. They want sheep.*	Family		
	*Never trade liberty for safety.*	Rest and relaxation		
**Appeal feedback (n=6, 9%), comment shared feedback on the design of the message appeal itself**				
	*Hmm my family has never stopped how did we survive?*	Return to normalcy		
	*Our freedom was already paid for from the blood of our fathers and their fathers before them....we don't need your permission.*	Freedom		

In addition, some comments provided feedback on the message appeals (n=6, 9%). One comment interpreted the *Family* message appeal as a fear tactic by stating, “coercion bully fear tactics have got to stop.” For the *Return to normalcy* message appeal, 1 comment mentioned that life never changed due to the pandemic by stating “hmm my family has never stopped how did we survive?” For the *Lifestyle* message appeal, 1 comment expressed confusion about the meaning by asking, “…are you going to give somebody a dream vacation if they get the jab now…” For the *Freedom* appeal, 1 comment portrayed disapproval toward equating freedom with the COVID-19 vaccine by stating, “our freedom was already paid for from the blood of our fathers and their fathers before them…we don’t need your permission.”

### Advertising Budget

Facebook Ad Manager’s CBO feature allocated more funds to message appeals resonating well with the target audience based on the identified goal (Reach). The project had a budget of US $26,600.00 (Table 7). Facebook allocated the most money (US $15,537.11) toward the *Family* message appeal due to its high performance. The *Return to normalcy* message appeal received the second-highest budget allocation (US $6083.52).

**Table 7:** CPM^a^ and CPC^a^ metrics for Message appeals.

Message appeals	Amount spent (US $)	CPM (US $)	CPC (US $)
Family	15,537.11	3.44	5.10
Return to normalcy	6083.52	3.47	5.56
Lifestyle	1748.46	3.40	5.64
Freedom	1409.73	3.37	4.93
Rest and relaxation	1066.32	3.32	5.83
Social connectedness	754.86	3.37	7.40
Total	26,600.00	3.43	5.29

^a^See Textbox 1 for definitions.

Since Reach was the primary objective, CPM was the most important metric. It evaluated how much of the advertising budget was required to deliver 1000 impressions based on the message appeal. The *Rest and relaxation* message appeal had the lowest CPM (US $3.32) among all 6 message appeals. While the *Family* and *Return to normalcy* message appeals had the highest performance across reach, engagement, and clicks, these message appeals also had the highest CPMs. The *Family* message appeal cost US $3.44 for 1000 impressions. The *Return to normalcy* message appeal cost US $3.47 for 1000 impressions. All 6 message appeals had a CPM of less than US $4.

While clicks were not the primary objective, the CPC metrics still provided valuable insight into how much of the advertising budget was required to get a person to visit www.vaccines.gov based on the message appeal. The *Freedom* message appeal had the lowest CPC of US $4.93. The *Family* and *Return to normalcy* message appeals had the next lowest CPCs of US $5.10 and US $5.56, respectively. The *Lifestyle*, *Rest and relaxation*, and *Social connectedness* message appeals had CPCs of US $5.64, US $5.83, and US $7.40, respectively.

## Discussion

### Principal Findings

This evaluation identified message appeals on parental concerns and sentiments that were most successful at exposing COVID-19 vaccination messages, specifically about travel, to millennial parents (25 to 40 years old) using Facebook Ads Manager. Overall, the *Family* and *Return to normalcy* message appeals reached the greatest number of people and garnered the most impressions out of all 6 message appeals. The *Family* and *Return to normalcy* message appeals also received the greatest number of engagements, respectively. On October 29, 2021, the Food and Drug Administration (FDA) authorized the Pfizer-BioNTech COVID-19 vaccine for emergency use in children 5-11 years [18], which likely impacted the performance of the message appeals.

The majority of comments portrayed negative opinions about COVID-19 vaccination, which conflicts with the overwhelmingly positive sentiment expressed through the post reactions. Because COVID-19 vaccination has become a controversial topic in politics, it was observed that any message that mentions the vaccine, regardless of the appeal, was used as an outlet to vent negative emotions. Therefore, the majority of the comments did not provide valuable insight for analyzing how these vaccine messages were framed. Upon evaluation of all message appeals, the *Family* and *Return to normalcy* message appeals highlighted the utility of creating COVID-19 vaccination messages that address parental concerns on how to keep their families safe during the pandemic instead of creating COVID-19 vaccination messages based on their sentiments. Evaluation of social media metrics are discussed below.

### Reach

The Reach and Impressions metrics revealed how successful each message appeal was at exposing the COVID-19 vaccination message to the target audience. The *Family* appeal reached 3,572,140 people (53.96%) and delivered 4,515,836 impressions (58.28%), the highest reaching message appeal. According to Moore et al [19], extrinsic motivators such as protecting one’s family and community was reported as a driving motivator for the COVID-19 vaccination among hesitant adopters of the vaccine. Additionally, Evans et al [20] conducted interviews with Australian parents on their experiences with the COVID-19 vaccine. In those interviews, parents consistently brought up the importance of protecting their children and keeping them safe. The *Family* appeal in this effort was the only message appeal that directly mentioned *kids* in the text component, while the other appeals referred to *family* in general*.* Therefore, it is plausible that this message appeal resonated most with a parent’s duty to protect their children. In addition, the FDA authorization of the COVID-19 vaccine for children 5-11 years occurred during this time. Therefore, the performance of the *Family* appeal was likely impacted.

The *Return to normalcy* message appeal reached 1,639,476 (24.77%) people and delivered 1,754,227 (22.64%) impressions, the second-highest reaching message appeal. Returning to a normal pre–COVID-19 lifestyle was identified as a main intrinsic motivator to get the COVID-19 vaccination among hesitant adopters of the vaccine [19]. Additionally, Bell et al [21] conducted a study to investigate the views of parents and guardians living in England with young children on the COVID-19 vaccine. The parents and guardians emphasized the need to return back to normal due to the unsustainable nature of lockdown and its physical and mental harm on children’s educational and social development. Therefore, it is understandable that the *Return to normalcy* message appeal reached the second highest number of people after the *Family* appeal.

While all message appeals were developed based on reported sentiments and concerns from parents during the COVID-19 pandemic (Table 1), the other 4 message appeals (*Lifestyle*, *Freedom*, *Rest and relaxation*, and *Social connectedness*) did not directly address the concerns of a parent. Instead, they focused on motivating parents to get vaccinated based on individual pandemic-specific sentiments. These sentiments included longing for a vacation (*Lifestyle*), needing a break from their daily home lives (*Freedom*), escaping life’s stresses (*Rest and relaxation*), and reconnecting with loved ones (*Social connectedness*). This finding highlights the utility of creating COVID-19 vaccination messages that address parental concerns, particularly related to their children, over motivating parents based on their own sentiments.

### Clicks and Click-Through Rate

The clicks directing users to www.vaccines.gov provided helpful insight into how successful the message appeal was at motivating users to search for more information about the COVID-19 vaccination. The CTR metric also provided context to how successfully each message appeal directed users to the website based on how many times the message appeal was seen. The *Family* message appeal was clicked on 3049 times (60.68%, CTR=0.06%), and the *Return to normalcy* message appeal was clicked on 1095 times (21.79%, CTR=0.06%). According to a report on industry-specific Facebook Ads benchmarks, the CTR benchmarks for the health and medical field was 0.83% and for the travel and hospitality field was 0.90% [22]. Because the Reach metric was identified as the Result, the CBO feature prioritized delivering the message appeals to the highest number of people over the highest number of clicks, resulting in CTRs lower than industry benchmarks.

### Sentiment

The Post reactions and Comments provided both quantitative and qualitative feedback on how users felt about the message appeals. Upon analyzing the 289 post reactions, 48.10% (n=139) were positive, 43.60% (n=126) were neutral or ambiguous, and 8.30% (n=24) were negative. The *Family* message appeal received the highest number of positive reactions (n=82, 28.37%), with the *Return to normalcy* message appeal receiving the next highest (n=32, 11.07%). Since both message appeals framed COVID-19 vaccination as a solution to real parental concerns, it was observed that these message appeals received positive interactions. The *Lifestyle* (n=7, 2.42%), *Freedom* (n=8, 2.77%), *Rest and relaxation* (n=5, 1.73%), and *Social connectedness* (n=5, 1.73%) message appeals focused on parental sentiments and framed COVID-19 vaccination using an aspirational, escapist method. Therefore, parents might have had a more challenging time relating to or positively connecting with these messages since they were not grounded in a parent’s reality during the COVID-19 pandemic.

Most of the 67 comments were negative (n=46, 68.66%), while a majority of the post reactions expressed a positive sentiment (n=139, 48.10%). It was also observed that messages that mentioned the vaccine, regardless of the appeal, were also used as an outlet to vent negative sentiments toward politics (n=13, 19.40%). Therefore, several comments did not provide valuable insight for analyzing how these vaccine messages were framed.

However, some comments provided insight to understand how some members of the target audience interpreted a message appeal. For instance, the comment “hmm my family has never stopped how did we survive?” on the *Return to normalcy* message appeal demonstrated the importance of designing public health messages grounded in the target audience’s actual behaviors rather than the expected or desired behaviors of the target audience. The *Return to normalcy* message appeal would resonate with people who adopted COVID-19 prevention behaviors such as physical distancing, wearing masks, and testing. However, if people did not change their lifestyles due to the pandemic, this message appeal would not resonate. In addition, 1 comment on the *Freedom* message appeal (“our freedom was already paid for from the blood of our fathers and their fathers before them…we don’t need your permission”) interpreted freedom in a partisan way as opposed to the intended escapist way. Overall, the post reactions provided a solid overview of the sentiment for the message appeal, and comments provided insight into how the message appeal was interpreted.

### Advertising Budget

Previous public health campaigns on Facebook with similar target audiences can provide appropriate benchmarks to evaluate how an advertising budget was spent. Therefore, the CPMs for other public health campaigns that targeted parents with young children were calculated for comparison purposes (Table 8). A lower CPM is preferred to maximize the number of impressions with the set advertising budget.

**Table 8:** Previous public health campaign CPMs^a^.

Public health campaign	Advertising budget (US $)	Impressions	CPM (US $)
Arshanapally et al [8] organized a Facebook campaign to disseminate messages on children’s development to parents with young children.	11,750	2,434,320	4.83
Skeens et al [23] used Facebook to recruit parents and their school-aged children for a study examining impact of COVID-19 on families.	2280	213,120	10.70
Cho et al [24]^b^ conducted a Facebook campaign to recruit parents and children with advanced cancer for an intervention study.	120,000	34,839,472	3.44

^a^See Textbox 1 for definitions.

^b^Cho et al [24] used a budget of US $120,000 for years 1 and 2 of their campaign. Therefore, all metrics only reflect years 1 and 2.

All message appeals, except the *Return to normalcy* appeal, had the same or lower CPMs than the CPM benchmarks set by other similar public health campaigns in Table 8 (*Family*: US $3.44; *Return to normalcy*: US $3.47; *Lifestyle*: US $3.40; *Freedom*: US $3.37; *Rest and relaxation*: US $3.32; *Social connectedness*: US $3.37). The *Return to normalcy* appeal was only slightly higher (+US $0.03) than the CPM benchmark set by Cho et al [24]; however, it remained lower than the CPM benchmarks set by the public health campaigns organized by Arshanapally et al [8] and Skeens et al [23].

Despite being among the lowest for reach and engagement performance, the *Freedom* (CPM=US $3.37) and *Rest and relaxation* (CPM=US $3.32) message appeals cost the least to disseminate the COVID-19 vaccination message to the target audience.

### Limitations

First, the text components of the message appeals were not isolated from the images. Therefore, it is unknown whether the images played a significant role in how users interacted with the message appeals or vice versa. Second, only the Facebook advertising budget was included in this evaluation. Other costs such as staffing, graphic design, and campaign management needed to execute this project were not included. Third, the authors did not survey users on Facebook for feedback on the message appeals. Therefore, the authors could only use existing literature, existing benchmarks, and previous public health campaigns to draw conclusions on why certain message appeals performed better on Facebook than others. Fourth, this project occurred in a constantly changing COVID-19 landscape where vaccine authorization was ongoing and public health recommendations were evolving. For instance, on October 29, 2021, the FDA authorized the Pfizer-BioNTech COVID-19 vaccine for emergency use in children 5-11 years (FDA), which likely impacted the performance of the message appeals. Therefore, external factors could not be controlled for.

### Conclusions

This evaluation establishes new benchmarks for future public health campaigns targeting parents as well as contributes to the public health communication literature by reporting which message appeals were the most successful at exposing COVID-19 vaccination messages, specifically about travel, to millennial parents (25 to 40 years old) using Facebook Ads Manager. Health communicators can use travel, specifically the *Family* and *Return to normalcy* message appeals, to reach parents in their future COVID-19 vaccination campaigns. Public health programs can also utilize the lessons learned from this evaluation to communicate COVID-19 information to their parent populations.

## Abbreviations

CBO: Campaign Budget Optimization
CDC: Centers for Disease Control and Prevention
CPC: cost-per-click
CPM: cost-per-mille
CTR: click-through rate
FDA: Food and Drug Administration

## Data Availability

All data generated or analyzed during this study are included in this published paper.
